# A merged microarray meta-dataset for transcriptionally profiling colorectal neoplasm formation and progression

**DOI:** 10.1038/s41597-021-00998-5

**Published:** 2021-08-11

**Authors:** Michael Rohr, Jordan Beardsley, Sai Preethi Nakkina, Xiang Zhu, Jihad Aljabban, Dexter Hadley, Deborah Altomare

**Affiliations:** 1 Burnett School of Biomedical Sciences, College of Medicine, University of Central Florida, Orlando, FL USA; 2 Department of Medicine, University of Wisconsin Hospital and Clinics, Madison, WI USA; 3 Department of Clinical Sciences, College of Medicine, University of Central Florida, Orlando, FL USA

**Keywords:** Cancer genomics, Microarrays

## Abstract

Transcriptional profiling of pre- and post-malignant colorectal cancer (CRC) lesions enable temporal monitoring of molecular events underlying neoplastic progression. However, the most widely used transcriptomic dataset for CRC, TCGA-COAD, is devoid of adenoma samples, which increases reliance on an assortment of disparate microarray studies and hinders consensus building. To address this, we developed a microarray meta-dataset comprising 231 healthy, 132 adenoma, and 342 CRC tissue samples from twelve independent studies. Utilizing a stringent analytic framework, select datasets were downloaded from the Gene Expression Omnibus, normalized by frozen robust multiarray averaging and subsequently merged. Batch effects were then identified and removed by empirical Bayes estimation (ComBat). Finally, the meta-dataset was filtered for low variant probes, enabling downstream differential expression as well as quantitative and functional validation through cross-platform correlation and enrichment analyses, respectively. Overall, our meta-dataset provides a robust tool for investigating colorectal adenoma formation and malignant transformation at the transcriptional level with a pipeline that is modular and readily adaptable for similar analyses in other cancer types.

## Abstract (summary)

**Table:** 

Measurement(s)	transcriptome • colorectal cancer
Technology Type(s)	microarray
Factor Type(s)	gene expression
Sample Characteristic - Organism	Homo sapiens

Machine-accessible metadata file describing the reported data: 10.6084/m9.figshare.14589006

## Background & Summary

Throughout the past decade, bioinformatics-based analyses have become a popular means for testing *in vitro* and *in vivo* results against data from human tissue samples vis-à-vis publicly accessible microarray and RNAseq datasets. This has been especially true in the cancer research field, which has leveraged a growing amount of available data from repositories such as the Gene Expression Omnibus (GEO)^1^, ArrayExpress^2^, and cBioPortal^3^ to facilitate pre-clinical modelling, delineate novel pathways involved in tumorigenesis, and discover clinically-relevant biomarkers. When such data is used in conjunction with third-party software such as Gene Set Enrichment Analysis (GSEA)^4^ and Ingenuity Pathway Analysis (IPA)^5^, transcriptome-wide analysis can provide a powerful tool for generating and testing hypotheses. However, because analytic performance depends on the quality and quantity of tissue samples, a plurality of investigations preferentially utilize datasets supplied by The Cancer Genome Atlas (TCGA) as they have been extensively validated and are robust in terms of sample number and included clinicopathology meta-data. Despite this, many TCGA datasets remain unsuitable for more specialized areas of cancer research. For example, tracking neoplasm development and progression using *in silico* approaches is constrained due to the lack of pre-malignant sample representation within TCGA datasets, a fact that is especially apparent for colorectal cancer (CRC)-related research^6^.

CRC serves as an exemplary model for investigating neoplastic progression as molecular events contributing to adenoma formation and progression are well described^7^ and are readily testable *in vitro* and *in vivo*^8^. Although much focus has been directed towards dissecting mechanisms related to genomic alterations, a paradigm shift has occurred in the form of transcriptional profiling for elucidating key drivers and suppressors of early tumorigenesis. The relative ease of tissue acquisition combined with the development of more reliable and cost-effective array platforms has contributed to the considerable rise in the number of publicly available datasets containing adenoma samples. However, this rise has resulted in the publication of many discordant results for essentially the same underlying biological process, thereby posing major challenges for establishing consensus^9^. Because inter-study heterogeneity caused by differences in study design, sample preparation, patient cohorts, and choice of array platform (amongst others) further complicates this process, using conventional meta-analytic techniques such as random-effects modelling to generate consensus has proven insufficient as results are limited to gene-level summaries^10^. In contrast, merging pre-processed datasets followed by batch correction and gene filtering effectively enables more complex meta-transcriptomic analyses, even demonstrating results comparable in robustness to TCGA datasets in terms of included clinicopathologic meta-data^10–12^.

**Fig. 1:**
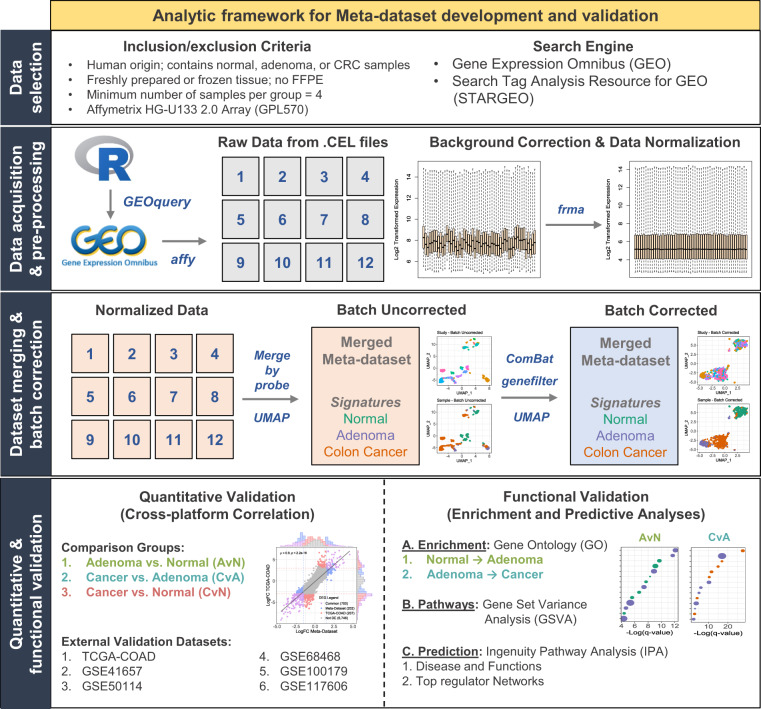
Study Design. Schematic detailing data selection, acquisition, pre-processing, merging, and technical validation.

Here, we developed a merged Meta-dataset containing 231 normal, 132 adenoma, and 342 colon cancer tissue samples across twelve independent studies to serve as a central compendium for *in silico* modelling and bioinformatic analyses of colorectal neoplastic progression. The overall study design including our pipeline and technical validation is outlined in Fig. 1. We implemented a modified analytic framework based on a previously established workflow^13^ to enable Meta-dataset construction without the use of the *inSilicoDB* R package. Briefly, microarray studies of the same chip platform (GPL570) and annotation package (hgu133plus2) meeting our inclusion criteria were identified using the Search Tag Analysis Resource for GEO (STARGEO) as a search proxy for GEO^14^. Pre-processing included downloading raw data from GEO followed by background correction, expression normalization, and log2 transformation via frozen Robust Multiarray Averaging (fRMA)^15^. The Meta-dataset was then generated by merging all pre-processed datasets by matching probe sets and then batch corrected using the empirical Bayes estimation method, or ComBat^9^. Thereafter, low variant probes were filtered from the Meta-dataset to facilitate technical validation and downstream differential expression analysis. We implemented two metrics to validate the reliability, accuracy, and robustness of the Meta-dataset. First, quantitative validation was performed via cross-platform correlation of gene trends with the TCGA colon adenocarcinoma (COAD) dataset^6^ and five external GEO datasets for all pairwise comparisons corresponding to normal versus adenoma (AvN), CRC versus adenoma (CvA), and CRC versus normal (CvN) signatures. Differentially expressed genes were then compared to four additional studies to assess overlap and consistency. Second, all signatures were functionally validated through integrative use of functional enrichment analyses using gene ontology (GO), gene set variance analysis (GSVA), and IPA with results being compared internally and to the literature.

Ultimately, our Meta-dataset represents a centralized dataset for studying early colorectal neoplasm dynamics with a straightforward workflow that is readily adaptable for other types of cancer.

## Methods

### Selection of microarray studies

To minimize the number and impact of batch effects, Minimum Information About a Microarray Experiment (MIAME)-compliant microarray studies sourced from GEO were selected based on predefined inclusion/exclusion criteria including: A) having either freshly prepared or frozen human tissue samples, B) a minimal number of four samples per tissue type, C) use of the GPL570 platform (Affymetrix Human Genome U133 Plus 2.0 array) for matching probe sets, and D) includes two of either normal, adenoma, or CRC tissue samples (Fig. 1). To ensure a robust number of probes and genes are represented in the Meta-dataset, studies utilizing base hgu133-based platforms (GPL96 and GPL97) were not included as the transcriptome coverage is substantially less than that provided by GPL570 despite the use of identical probes. We then used the STARGEO tool developed by our collaborator^14^, to efficiently parse sample characteristics to identify studies meeting these criteria. Overall, twelve independent studies were chosen out of an initial list of 256 to be used in the construction of the Meta-dataset (Table 1).

**Table 1:** List of studies meeting our inclusion/exclusion criteria used for Meta-dataset construction and technical validation.

Datasets used for Meta Dataset construction		
Accession #	Samples	Platform
GSE4183	38	GPL570, [HG-U133_Plus_2] Affymetrix Human Genome U133 Plus 2.0 Array
GSE8671	64	GPL570, [HG-U133_Plus_2] Affymetrix Human Genome U133 Plus 2.0 Array
GSE9348	82	GPL570, [HG-U133_Plus_2] Affymetrix Human Genome U133 Plus 2.0 Array
GSE15960	18	GPL570, [HG-U133_Plus_2] Affymetrix Human Genome U133 Plus 2.0 Array
GSE20916	125	GPL570, [HG-U133_Plus_2] Affymetrix Human Genome U133 Plus 2.0 Array
GSE21510	44	GPL570, [HG-U133_Plus_2] Affymetrix Human Genome U133 Plus 2.0 Array
GSE22598	34	GPL570, [HG-U133_Plus_2] Affymetrix Human Genome U133 Plus 2.0 Array
GSE23194	17	GPL570, [HG-U133_Plus_2] Affymetrix Human Genome U133 Plus 2.0 Array
GSE23878	59	GPL570, [HG-U133_Plus_2] Affymetrix Human Genome U133 Plus 2.0 Array
GSE32323	34	GPL570, [HG-U133_Plus_2] Affymetrix Human Genome U133 Plus 2.0 Array
GSE33113	96	GPL570, [HG-U133_Plus_2] Affymetrix Human Genome U133 Plus 2.0 Array
GSE37364	94	GPL570, [HG-U133_Plus_2] Affymetrix Human Genome U133 Plus 2.0 Array
Total	705	
**Datasets used for technical validation**		
GSE41657	88	GPL6480, Agilent-014850 Whole Human Genome Microarray 4x44K G4112F
GSE50114	46	GPL6480, Agilent-014850 Whole Human Genome Microarray 4x44K G4112F
GSE68468	387	GPL96, Affymetrix Human Genome U133A Array
GSE100179	60	GPL17586, [HTA-2_0] Affymetrix Human Transcriptome Array 2.0
GSE117606	208	GPL25373, [HT_HG-U133_Plus_PM] Affymetrix HT HG-U133 + PM Array Plate
TCGA-COAD	519	Illumina HiSeq

Sample number and array platforms are provided for each study.

### Data acquisition and pre-processing

Raw data contained in zipped .TAR packages were downloaded from GEO using the *getGEOSuppFiles* function in the *GEOquery* (version 2.58.0) R package^16^. Individual .CEL files for each study were unpacked using the *untar* function in base R, loaded using the *ReadAffy* function part of the *affy (*version 1.68.0) package^17^, and subsequently background corrected and log-transformed by frozen Robust Multiarray Averaging (fRMA) using the *frma* (version 1.42.0) package^15^. Compared to traditional RMA, *fRMA* utilizes pre-computed probe variances to normalize raw microarray data and was shown to outperform RMA when pre-processing individual datasets for grouped analyses^15^.

### Meta-dataset construction

Following fRMA normalization, arrays from individual datasets were merged by matching probe.s Interstudy batch effects were identified by Uniform Manifold Approximation and Projection (UMAP) using the *umap* (version 0.2.7.0) package^18^ and removed using the original parametric iteration of *ComBat* within the *sva* (version 3.38.0) package^19^. UMAP was used over traditional principal component analysis (PCA) to identify batches due to its ability to better represent local relationships whilst preserving global structure, thereby accentuating non-biological batch clusters for rapid identification and confirmation of their removal post-processing^18^. For batch correction, ComBat was chosen due to its flexibility, reliability, and ability to set covariates of interest. Because ComBat assumes that differences in batches are non-biological^9^, biological covariates corresponding to normal, adenoma, and colon cancer samples can be specified and preserved to prevent over-normalization and loss of natural variance; a feature that was readily validated by UMAP.

Next, probes with an expression variance in the lower 75^th^ percentile were filtered from the Meta-dataset using the *genefilter* function in the *oligo* (version 1.54.1) package^20^. Previous reports have suggested the utility of filtering datasets for low variant probes, especially for differential expression (DE) analysis as significance arising from low variance and not magnitude of change impedes meaningful data interpretation^21^. Finally, redundant probes were collapsed to their corresponding human gene symbol by maximum average expression using the *hgu133plus2.db* (version 3.2.3) package. Collectively, the constructed Meta-dataset contains a total of 705 samples including 231 normal, 132 adenoma, and 342 CRC tissue samples across 12 independent studies. A complete list of clinical and histological meta-data can be found in Table 2.

**Table 2:** Clinicopathologic information.

Gender		Average Age	Tissue Sample		Clinicopathology		Metastasis/ Recurrence		Anatomical Location	
Male	179	66.54 ± 11.72	Normal	31	Pathologic Stage II	42	M0	65	Cecum	5
			Adenoma	27			M1	9	Ascending Colon	4
			CRC	121			Recurrence	8	Transverse Colon	8
									Descending Colon	1
									Sigmoid	21
									Rectosigmoid	1
									Rectum	11
Female	194	66.19 ± 11.20	Normal	51	Pathologic Stage II	48	M0	58	Cecum	4
			Adenoma	23			M1	9	Ascending Colon	9
			CRC	120			Recurrence	10	Transverse Colon	1
									Descending Colon	7
									Sigmoid	21
									Rectosigmoid	1
									Rectum	11
Unidentified	332	n/a	Normal	149	Low Grade Polyp Dysplasia	16	M0	68	Cecum	0
			Adenoma	82	High Grade Polyp Dysplasia	13	M1	18	Ascending Colon	8
			CRC	101	Dukes A/B	14	Recurrence	26	Transverse Colon	2
					Pathologic Stage I	10			Descending Colon	8
					Pathologic Stage II	37			Sigmoid	32
					Pathologic Stage III	23			Rectosigmoid	0
					Pathologic Stage IV	18			Rectum	14

Breakdown of clinical and pathologic characteristics stratified by gender in the Meta-dataset. The number of samples (n) are given to the right of each variable.

### TCGA and other GEO datasets

Raw data from TCGA-COAD dataset was downloaded using the *TCGAbiolinks* (version 2.18.0) package^22^. Data was pre-processed by within-lane normalization using the “GC content” option which includes loess robust local regression followed by global scaling and quantile normalization^23^. Thereafter, genes with row averages less than 0.25 were filtered out of the dataset and results were returned as counts per million (CPM). Finally, the TCGA dataset was prepared for DE analysis using the *voom* function and annotated to human gene symbol from ensemble ID.

Data from GEO studies not using the GPL570 platform but still meeting the other inclusion criteria were also used for Meta-dataset validation and downloaded in their pre-processed state using the *getGEO* function in the *GEOquery* package. Redundant probes were collapsed and annotated to human gene symbols prior to analysis.

### UMAP, variance stabilization, and sample co-clustering analysis

UMAP was performed to identify batches, validate their removal, and ensure the preservation of biological signature post-batch correction. UMAP was run using twenty nearest neighbors for each for pre- and post-batch corrected data after which coordinates from the top two UMAP components were extracted to be visualized and color-coded either by study or sample using the *ggplot* function within the *ggplot2* (version 3.3.3) package^24^. To determine the overall effect of batch correction on the Meta-dataset, variance stability was compared between batch corrected and batch uncorrected Meta-datasets. Column (array) variances were determined using the *colVars* function within the *Rfast* (version 2.0.1) package^25^ and compared via boxplot to map cumulative distributions. Statistical analysis was performed using a Wilcoxon signed-rank test.

To assess the consistency of clustering results and its potential impact on downstream analyses, we performed unsupervised density-based consensus clustering^26^. Specifically, sample types were predicted based on their UMAP clustering coordinates from 1,000 bootstraps of the density-based UMAP (DBU) cluster algorithm^27^ within the *fpc* (version 2.2.9) package^28^ with the reachability distance epsilon (eps) set to 0.45 and reachability minimum number of points (MinPts) set to 5 (both determined empirically). Co-clustering was then determined by comparing the consensus sample predictions to the actual sample types and presented via confusion table.

### Cross-platform correlation analysis

Cross-platform correlation was used to quantitatively validate the Meta-dataset. Because gene expression results obtained from different array platforms cannot be directly compared, we opted instead for comparing the log2 fold change (LogFC) values, or expression trends of genes common between the Meta-dataset and six external datasets. Previous studies have shown this method to be a robust means of validation as global gene expression trends are generally preserved across tissue types despite sample and study heterogeneity^12,13^. In order to accomplish this, the LogFC of genes across adenoma versus normal (AvN), CRC versus adenoma (CvA), and CRC versus normal (CvN) comparison groups were computed using the *limma* (version 3.46.0) package. For comparing the Meta-dataset (microarray) with the TCGA-COAD dataset (RNAseq), the latter was voom-transformed prior to enumerating LogFC values. Cross-platform correlation between the LogFC values of common genes was then performed using the Spearman correlation coefficient to assess overall relationships.

### Differential expression (DE), Pathway Enrichment (PE), and Gene Ontology (GO) analysis

Differential expression (DE) analysis for AvN, CvA, and CvN comparison groups was carried out using *limma*. Specifically, DE analysis was independently performed on the top 25% most variable genes between each group. Genes were considered DE if they met the uniform threshold of having a |LogFC| ≥ 1.0 and False Discovery Rate (FDR) *q*-value < 0.01. Comparison of differentially expressed genes (DEGs) between each contrast was then visualized by a Venn-diagram using the *VennDiagram* (version 1.6.20) package^29^.

Pathway enrichment (PE) analysis for AvN, CvA, and CvN comparison groups was carried out by gene set variance analysis (GSVA) using the *GSVA* (version 1.38.2) package with default parameters^30^. Specifically, all C2 curated gene sets (*c2.all.v7.4.symbols*), which includes canonical pathways, KEGG, BIOCARTA, and REACTOME annotations, was downloaded from MSigDB and used for enumerating PE scores from the top 25% variable genes as before. A minimum gene set size was set to 10 genes while the maximum size was set to 1,000 genes. Differentially enriched pathways (DEPs), or those with a |LogFC| ≥ 0.25 and FDR *q*-value < 0.01, were identified using *limma*. As before, a Venn-diagram was used to visualize both common and unique pathways amongst each contrast.

Gene Ontology (GO) analysis was performed both as a means of validating enriched pathways and to visually represent up- and downregulated biological processes characteristic of adenoma formation and malignant transformation. DEGs identified in the AvN and CvA groups were compared to the full list of genes obtained after collapsing and annotating redundant probes. GO terms relating to biological processes were identified using the *topGO* (version 2.42.0) package^31^. Specifically, analysis was restricted to GO terms of more than 20 genes and statistical significance was determined by Fisher’s Exact test of gene ratios, or the number of observed enriched genes compared to the number of genes expected to be enriched by chance. Results were visualized as dot plots of the top 14 significant GO terms associated with up- and downregulated DEGs.

### Ingenuity pathway analysis (IPA)

Prediction-based IPA analysis was used to functionally validate the Meta-dataset in an unbiased way. IPA utilizes advanced literature search techniques from a curated database to predict regulators, mechanistic networks, and sample characteristics based upon the magnitudes and directions of DEG LogFC values. We therefore used IPA to predict characteristics and potential mechanisms of AvN, CvA, and CvN comparison groups to validate that our Meta-dataset indeed represents each sample and potentially their progression. To do this, the list of DEGs between each pairwise comparison determined previously were used for analysis.

First, three disease and function predictions and their corresponding FDR *q*-values (determined using Fisher’s Exact Test) were used to validate that the signatures corresponded to the correct tissue type and state. Afterwards, the top regulatory networks corresponding to adenoma formation (AvN) and malignant transformation (CvA) were assessed and visualized. Information from the regulatory network including which upstream regulator and downstream pathway was predicted to be active or inhibited was then compared both to the GO analysis (for determining internal consistency) and the literature (for assessing whether results are congruent with what is known).

## Data Records

The Meta-dataset and associated clinical meta-data data are available at ArrayExpress^32^. Datasheets used throughout the R code script to perform all the analyses can be found at figshare^33^. All dataset used in constructing the Meta-dataset as well as its technical validation can be found at GEO (https://www.ncbi.nlm.nih.gov/geo/) or Genomic Data Commons (https://portal.gdc.cancer.gov/) and include: GSE4183^34^, GSE8671^35^, GSE9348^36^, GSE15960^37^, GSE20916^38^, GSE21510^39^, GSE22598^40^, GSE23194^41^, GSE23878^42^, GSE32323^43^, GSE33113^44^, and GSE37364^45^, TCGA-COAD^6^, GSE41657^46^, GSE50114^47^, GSE68468^48^, GSE100179^49^, and GSE117606^50^.

## Technical Validation

### Data acquisition and pre-processing

#### Data selection

An analytic pipeline was used in conjunction with inclusion/exclusion criteria for selecting microarray datasets suitable for merging (Fig. 1). To ensure relative homogeneity of samples and reduce the multiplicative impact of batch effects, only studies using freshly prepared or frozen human tissue were selected. Formalin fixed paraffin embedded (FFPE) tissue samples were excluded due to the heterogeneity of microarray efficiency stemming from differing fixation protocols, low nucleic acid purity or degradation^51^. In addition, sessile serrated adenoma samples were excluded when possible. Furthermore, studies used to construct the Meta-dataset were restricted to those using the GPL570 Affymetrix platform to reduce inter-platform batch effects and enable probe-probe matching when merging. Although the rigor of this pipeline substantially reduced the total number of candidate studies, we believe minimization of batch effects was a justified trade-off.

#### Data normalization

**Fig. 2:**
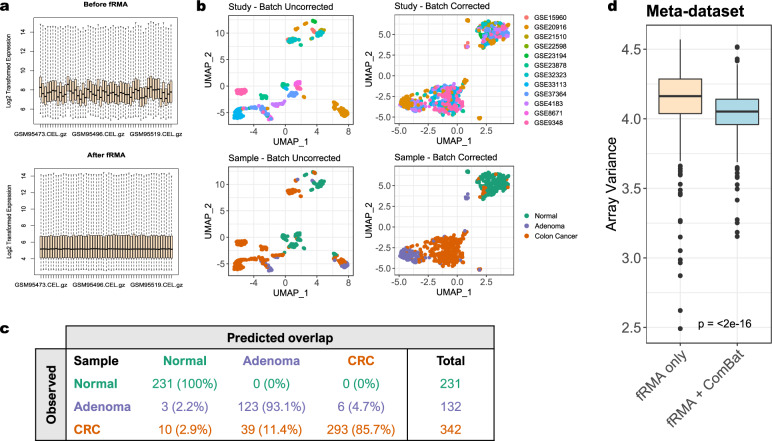
Data pre-processing and batch correction. (**a**) Boxplots showing global array expression distribution in GSE9348 before (top) and after (bottom) normalization using frozen Robust Multiarray Averaging (fRMA). (**b**) UMAP plots showing successful identification (left) and removal (right) of inter-study batch effects with preservation of normal, adenoma, and colon cancer biological signatures (bottom) through empirical Bayes estimation method (ComBat). (**c**) Confusion table comparing the co-clustering of each known sample type (rows) with their predicted clusters (columns) based on 1,000 bootstraps of the density-based UMAP algorithm. Both the number of samples and their percentage of total are provided. (**d**) Boxplot comparing the distribution of array variances between the batch uncorrected (fRMA only) and batch corrected (fRMA + ComBat) Meta-datasets. Statistical significance was determined using a Wilcoxon signed-rank test.

As explained previously, fRMA was chosen for normalizing raw microarray data due to its superior performance against conventional RMA for pre-processing datasets in batches prior to grouped analyses. For example, in the raw log2-transformed data from GSE9348^36^, substantial heterogeneity in global probe expression exists between patient microarrays (Fig. 2a, top). Application of fRMA greatly reduced noise and stabilized full-array expression ranges around the median (Fig. 2a, bottom). This procedure was applied to all datasets individually using the *frma* function prior to Meta-dataset construction.

#### Meta-dataset detailed information

Collectively, the Meta-dataset consists of 231 normal (32.8%), 132 adenoma (18.7%), and 342 colon cancer (48.5%) samples for a total of 705 samples. Based on the data available from the annotations provided by each of the 12 included studies, tissue from 179 males (25.4%), 194 females (27.5%), and 332 unidentified genders (47.14%) are represented. The overall mean age was 66.37 ± 11.46 y/o (Table 2). The average age for male subjects was 66.54 ± 11.72 y/o and 66.19 ± 11.20 y/o for female subjects, falling within the known average age of diagnosis for both genders (68 y/o males and 72 y/o females)^52^. Histopathologic data was more limited based on the studies utilized, including detailed information on 29 adenoma (16 low grade dysplasia and 13 high grade dysplasia) and 221 CRC samples (14 Dukes A/B, 10 Stage I, 37 Stage II, 23 Stage III, and 18 Stage IV). This also included 191 subjects being designated M0, 36 M1, and 44 subjects having recurrent disease. Finally, anatomical location was provided in some of the datasets resulting in the representation of 9 cecal, 21 ascending colon, 11 transverse colon, 16 descending colon, 74 sigmoid colon, 2 rectosigmoid, and 36 rectal samples. Information regarding other clinicopathologic characteristics and/or treatment status were not reported as they were not explicitly detailed in the meta-data of the studies.

### Batch identification and removal

#### Uniform Manifold Approximation and Projection analysis

UMAP was used to identify both non-biological and biological sources of variation. Specifically, the *umap* function was used with 20 nearest neighbors to enumerate the first two components explaining the greatest degree of variance from the pre- and post-batch corrected meta-datasets. Prior to batch correction, distinct clusters corresponding to both non-biological, or study-related batches (Fig. 2b, top left), and biological, or sample-related batches (Fig. 2b, bottom left), were identified. However, batch correction via ComBat resulted in a complete removal of non-biological batches (Fig. 2b, top right). Importantly, clusters corresponding to biological batches were preserved post-ComBat (Fig. 2b, bottom right), confirming that over-normalization did not occur and inherent differences between normal, adenoma, and CRC samples remained distinct. Moreover, we noted a degree of overlap (11.4%) between the adenoma and colon cancer clusters which was preserved post-batch correction and confirmed using unsupervised density-based clustering of the UMAP projection (Fig. 2c). This was an expected finding as expression changes in adenoma samples share more in common with CRC than normal epithelium, and serves as a secondary means of validation. Finally, due to the overall minimal amount of sample overlap, we did not isolate core samples for downstream analysis as doing so would effectively eliminate the impact of inherent tissue heterogeneity.

#### Effects of batch correction on array expression distribution

To assess the effect batch correction had on full array expression distribution, full-array expression variances between the batch uncorrected (fRMA only) and corrected (fRMA + ComBat) Meta-datasets were compared. As expected, batch correction significantly reduced (*P* < 0.0001) and stabilized array variances as reflected by compressed boxplot interquartile ranges (Fig. 2d).

### Validation of meta-dataset biological signatures

#### Quantitative cross-platform validation

Quantitative validation was performed by cross-platform correlation of common genes between the Meta-dataset and six external datasets of varying platforms detailed in Table 1. Because direct comparison of gene expression values across differing platforms is not possible, we opted instead for correlating gene trends between adenoma and normal samples (AvN), CRC and adenoma samples (CvA), and CRC and normal samples (CvA). This method has been used previously for confirming meta-dataset accuracy and robustness^13^. To accomplish this, redundant probes for all datasets were first collapsed to gene symbols using their respective annotation package followed by enumeration of log fold change (LogFC) values for each pairwise comparison using *limma*. LogFC values of genes common with the Meta-dataset were then correlated using Spearman correlation coefficient (*Rs*) to account for the potential of non-linear relationships. All results, including the number of samples for each comparison and the total number of correlated genes, are detailed in Table 3.

**Table 3:** Quantitative Validation.

Study	Platform	Samples (n)			Common Genes	Correlation of LogFC to Meta-dataset		
		Normal	Adenoma	Cancer		AvN	CvA	CvN
TCGA-COAD	Illumina Hi-Seq	41	0	478	7,856	-	-	0.90
GSE41657	Agilent 4 × 44K	12	51	25	7,578	0.72	0.60	0.74
GSE50114	Agilent 4 × 44K	9	37	0	5,979	0.88	-	-
GSE68468	Affymetrix HG U133A	0	13	374	5,912	-	0.78	-
GSE100179	Affymetrix HT Array 2.0	20	20	20	7,989	0.83	0.69	0.79
GSE117606	Affymetrix HT HG-U133+	65	69	74	7,752	0.79	0.73	0.84

Quantitative validation of the Meta-dataset was carried out by comparing the LogFC values of common genes across each pairwise comparison, including adenoma vs. normal (AvN), CRC vs. adenoma (CvA), and CRC vs. normal (CvN), between the Meta-dataset and six external datasets via Spearman correlation. In addition to the spearman correlation coefficient (*Rs*) given for each comparison, the instrument platform, the number of samples for each tissue type, and the number of common genes is provided. “-” indicates that no comparison was made due to the lack of tissue type representation. All *Rs* values are significant at *P* < 0.0001.

Overall, we observed strong gene trend correlations between our Meta-dataset and all external datasets with an average *Rs* of 0.81 ± 0.07 for AvN, 0.70 ± 0.08 for CvA, and 0.82 ± 0.07 for CvN groups. First, we noted that cross-platform correlation of CvA gene trends was the lowest across all studies. We believe that this is a result of more substantial tissue heterogeneity that exists for adenoma and CRC samples relative to healthy tissue. In contrast, we found that comparison of CvN gene trends produced the strongest correlations in almost all cases with the greatest association (*Rs* = 0.90) observed between the Meta-dataset (microarray) and voom-transformed TCGA-COAD (RNAseq) datasets, despite their vastly different approaches to expression profiling. This finding was not completely unwarranted as another study comparing merged microarray meta-datasets to the TCGA-LUAD and TCGA-LUSC lung cancer datasets observed cross-platform correlation values of 0.92 and 0.93, respectively^13^. In fact, we found that correlation performance was independent of platform type with no differences observed between Affymetrix- or Agilent-based platforms, providing further evidence of the robust nature and general applicability of our Meta-dataset.

#### Comparison of differentially expressed genes with other meta-datasets

To provide an additional degree of quantitative validation, we first identified DEGs, or genes with an absolute fold change of at least 2 (LogFC ≥ 1) and FDR *q-value* < 0.01 between AvN, CvA, and CvN comparison groups (Supplementary File 1), followed by cross-examination across four additional studies including three smaller CRC-related meta-datasets. For example, Dongmei *et al*. 2020 constructed a meta-dataset comprising only normal and CRC samples from 4 independent microarray studies and identified 10 key hub genes closely associated with the pathogenesis of CRC^53^. Of these, 9 (CDK4, CDH3, DKC1, UBE2S, UBE2C, GUCA2A, GUCA2B, TRIP13, and GTF3A) were DEGs in our Meta-dataset’s CvN group with the exception of EIF3B. Similarly, Xingjie *et al*. 2016 constructed a 3-study meta-dataset and identified 7 important hub genes contributing to the development of CRC^54^. As before, a majority (COL1A1, COL1A2, UGDH, ALDH1A1, FABP4, and MGLL) were differentially expressed in our Meta-dataset excluding MMP9.

To validate adenoma-based signatures, DEGs in the AvN and CvA groups were compared to those reported by Hauptman *et al*.^55^. This group not only created a 4-study meta-dataset representing all three sample types, but also provided a detailed list of DEGs for each pairwise comparison. We noted excellent concordance between our Meta-dataset with a 93% (127/137), 89% (23/26) and 100% (172/172) overlap of DEGs across AvN, CvA, and CvN contrasts, respectively. Finally, we opted to compare CvA DEGs with those identified by Druliner *et al*. 2019 as their investigation is one of only a handful providing insight into transcriptome-wide changes driving malignant transformation of adenomas^56^. This study is unique in that expression profiles of cancer-free and cancer-associated polyps were compared to identify genes directly linked with adenoma neoplastic progression. Overall, we found that a substantial number of these driver genes, including GREM1, CXCL5, PLAU, IGF1, IGF2, and EREG were identified as CvA DEGs in our Meta-dataset.

Collectively, the strong inter-platform correlation of gene trends combined with the high degree of DEG overlap across a variety of independent studies demonstrates the robust nature of our Meta-dataset and suggests that results obtained from in-depth analyses have the power and consistency necessary for pre-clinical modelling of CRC neoplastic progression.

#### Functional validation through integrative analyses

**Fig. 3:**
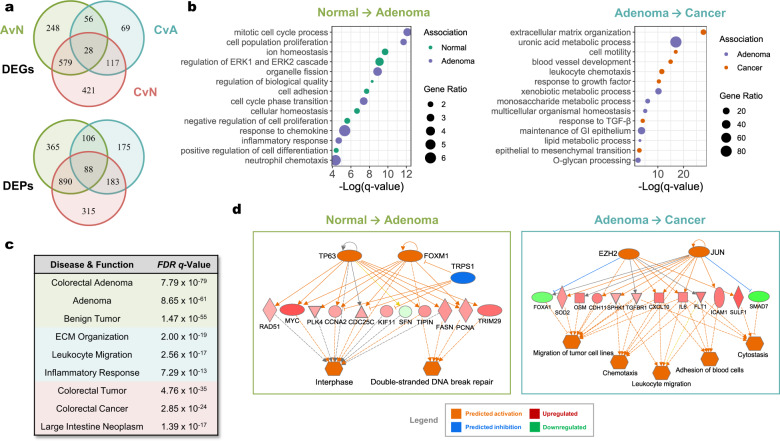
Functional validation. Functional validation of the Meta-dataset was carried out in three steps including comparison of differentially expressed genes (DEGs), functional enrichment, and prediction-based analyses. (**a**) Venn diagram comparing DEGs (|LogFC| ≥ 1.0 and FDR *q-value* < 0.01) (top) and differentially enriched pathways (DEPs; |LogFC| ≥ 0.25 and FDR < 0.01) (bottom) between AvN, CvA, and CvN contrasts showing the presence of genes and pathways that are unique to each phase along the neoplastic sequence. (**b**) Plots detailing representative enriched gene ontology (GO) terms corresponding to the early (normal to adenoma; AvN) (left) and late (adenoma to cancer; CvA) (right) phases of neoplastic progression. The color of each point is determined by its association to either tissue type based on whether the genes were down- or upregulated. The size of each point is determined by the gene ratio, or the ratio of significantly enriched genes to the expected number of genes by chance for each term. (**c**) Prediction-based validation of the AvN (green), CvA (blue), and CvN (red) signatures using IPA’s prediction of disease and function from the list of DEGs. (**d**) Top mechanistic networks corresponding to early (left) and late phases (right) of neoplastic progression determined by integrating IPAs prediction of upstream regulators (orange or blue) and direction of fold change of DEGs (red or green) showing strong agreement with functional enrichment results and what is known in the literature. All *p*-values were determined using Fisher’s Exact test followed by Benjamini-Hochberg correction for multiple comparisons.

In addition to quantitative validation, we also functionally validated AvN, CvA, and CvN signatures through integrative use of enrichment- and prediction-based analyses. This was done to provide both continuity and context to the quantitative validation results while also demonstrating the potential utility of our Meta-dataset for unearthing key genes, regulators, and pathways associated with early and late phases of colorectal neoplastic progression. Because transcriptome-wide changes are expected to be unevenly distributed along the neoplastic progression axis, we first investigated the degree of overlap between DEGs of each signature (Fig. 3a, top). Of the 1,318 total DEGs, 738 (48.6%) were unique to each signature, 752 (49.5%) were common between two groups, and only 28 (1.9%) were common between all three groups. Specifically, there were 827 DEGs uniquely involved in adenoma formation (AvN), 186 DEGs involved in malignant transformation (CvA), and 84 DEGs involved in both processes, suggesting that transcriptome-wide changes are more prominent during early phases of neoplastic progression.

To provide a functional context to these changes, we performed Gene Set Variance Analysis (GSVA) on the top 25% variable genes for each comparison group as well as Gene Ontology (GO) on DEGs. GSVA scores measures gene set enrichment variation across the entire dataset and provides sample-level enrichment scores based on the single sample Gene Set Enrichment Analysis (ssGSEA) algorithm, enabling accurate identification of differentially enriched pathways (DEPs) using linear modelling techniques such as *limma*^30^. Likewise, we assessed the degree of overlap of DEPs which were defined as |LogFC ≥ 0.25|and FDR *q-value* < 0.01 (Fig. 3a, bottom). We found that out of 2,122 DEPs, 815 (38.4%) were unique to each signature, 1,179 (55.6%) were common to two groups, and 88 (4.0%) were shared between all three groups (Supplementary File 2). In addition, 1,255 DEPs were unique to adenoma formation, 358 DEPs were unique to malignant transformation, and 194 DEPs were associated with all phases of progression. Of note, the top pathway positively associated with adenoma formation was SABATES_COLORECTAL_ADENOMA_UP (LogFC = 0.95, FDR = 1.90 × 10^−164^) while the top negatively associated pathway was SABATES_COLORECTAL_ADENOMA_DN (LogFC = −0.93, FDR = 1.69 × 10^−154^), both of which are derived from GSE8671^35^. Moreover, well known culprits of early neoplastic progression including pathways associated with epithelial-mesenchymal transition (EMT), DNA damage and repair, MYC activation, and hyperproliferation via cell cycle transition are AvN-associated DEPs. In the same light, pathways known to be closely associated with CRC pathogenesis such as TGFβ1^57^, FOXM1^58^, MYC^59^, angiogenesis and extracellular matrix (ECM) remodelling^60^ are CvA and CvN-associated DEPs. Importantly, DEPs were recapitulated by GO analysis of DEGs and collectively show that adenoma formation is marked by loss of cellular functions characteristic of differentiated tissue as well as hyperproliferation in response to genomic stress and potentially inflammation (Fig. 3b, left). In contrast, malignant transformation is defined by a loss of specialized metabolic functions with stark activation of ECM remodelling, inflammation, angiogenesis, and EMT (Fig. 3b, right).

Finally, we utilized prediction-based analysis via Ingenuity Pathway Analysis (IPA) software to identify key sample characteristics and mechanistic pathways as a secondary functional validation metric. IPA is a powerful tool that utilizes a curated database of scientific literature to predict regulators, pathways, and associated disease and functions from a list of genes and their corresponding LogFC and/or significance level^5^. To functionally validate AvN, CvA, and CvN signatures, we predicted associated disease and functions from DEGs. The analysis was based on the direction of the LogFC and FDR values and was restricted to drawing direct relationships from the human database. IPA correctly identified the adenoma signature from the AvN DEG list with Colorectal Adenoma (FDR = 7.79 × 10^−79^), Adenoma (FDR = 8.65 × 10^−61^), and Benign Tumor (FDR = 1.47 × 10^−55^) being top results (Fig. 3c). This was also true regarding the CRC signature, with Colorectal Tumor (FDR = 4.76 × 10^−35^), Colorectal Cancer (FDR = 2.85 × 10^−24^), and Large Intestine Neoplasm (FDR = 1.39 × 10^−17^) being predicted from the CvN DEG list. Top disease and functions associated with the CvA signature were in line with functional enrichment results. For mechanistic networks, we found that adenoma formation was associated with the activation of known CRC oncogenes FOXM1^58^ and TP63^61^, which was predicted to be primary regulators governing the activation of interphase cell cycle transition and DNA repair pathways (Fig. 3d, left). On the other hand, malignant transformation was characterized by activation of EZH2^62^ and JUN oncogenes^63^, which was predicted to enhance tumor cell migration and cytostasis (a known feature of EMT)^64^ as well as leukocyte chemotaxis and adhesion, presumably culminating in tumor infiltration (Fig. 3d, right). Importantly, our results mirrors what is known about early and late phases of neoplastic progression^65^ while also providing a wealth of knowledge that could shed light on less-described genes and/or pathways. Ultimately, we provide compelling evidence supporting the accuracy of our Meta-dataset and potential use as a powerful investigational tool for *in silico* modelling of colorectal neoplastic progression.

In the present study, we aggregated 705 arrays across 12 independent studies to create a Meta-dataset of normal, adenoma, and colon cancer samples for the primary goal of studying colorectal neoplasm formation and progression. Collectively, results from transcriptional profiling of early and late stages of neoplastic progression using our Meta-dataset not only demonstrated results that were generalizable across a variety of studies and array platforms, but also strongly agreed with the literature, thereby validating its accuracy and robustness. Moreover, by containing a breadth of adenoma samples our Meta-dataset provides distinct advantages over the conventional TCGA-COAD dataset, especially for investigating pre-malignant phases of colorectal neoplastic progression. It is our belief that this Meta-dataset provides a powerful public tool to facilitate further in-depth *in silico* analyses, biomarker discovery, pre-clinical modelling, and even hypothesis generation and testing. Of course, by making our Meta-dataset openly accessible, we invite the scientific community to apply novel tools and techniques to further dissect mechanisms associated with adenoma formation and malignant transformation.

## Supplementary information

Supplementary File 1

Supplementary File 2

## Data Availability

The R code used to construct and validate the Meta-dataset is available at Data Citation 2. Analyses were executed in R within the R Studio desktop (version 1.1.1103) suite. Microsoft’s open R version 4.0.2 (https://mran.microsoft.com/open) was used to take advantage of a multicore system to improve multithreaded processes and reduce computation time.

## References

[1] Edgar R (2002). Gene Expression Omnibus: NCBI gene expression and hybridization array data repository. Nucleic Acids Research.

[2] Athar A (2019). ArrayExpress update - from bulk to single-cell expression data. Nucleic Acids Res.

[3] Cerami E (2012). The cBio cancer genomics portal: an open platform for exploring multidimensional cancer genomics data. Cancer Discov.

[4] Subramanian A (2005). Gene set enrichment analysis: a knowledge-based approach for interpreting genome-wide expression profiles. Proc Natl Acad Sci USA.

[5] Krämer A, Green J, Pollard J, Tugendreich S (2014). Causal analysis approaches in Ingenuity Pathway Analysis. Bioinformatics.

[6] Cancer Genome Atlas Network. Comprehensive molecular characterization of human colon and rectal cancer. *Nature* **487**, 330–337 (2012) 10.1038/nature11252 PMC3401966 22810696

[7] Manne U, Shanmugam C, Katkoori VR, Bumpers HL, Grizzle WE (2010). Development and progression of colorectal neoplasia. Cancer Biomark.

[8] Johnson RL, Fleet JC (2013). Animal models of colorectal cancer. Cancer Metastasis Rev.

[9] Johnson WE, Li C, Rabinovic A (2007). Adjusting batch effects in microarray expression data using empirical Bayes methods. Biostatistics.

[10] Walsh C, Hu P, Batt J, Santos C (2015). Microarray meta-analysis and cross-platform normalization: Integrative genomics for robust biomarker discovery. Microarrays.

[11] Lim SB, Tan SJ, Lim W-T, Lim CT (2017). An extracellular matrix-related prognostic and predictive indicator for early-stage non-small cell lung cancer. Nat Commun.

[12] Lim SB, Tan SJ, Lim W-T, Lim CT (2019). Compendiums of cancer transcriptomes for machine learning applications. Sci Data.

[13] Lim SB, Tan SJ, Lim W-T, Lim CT (2018). A merged lung cancer transcriptome dataset for clinical predictive modeling. Sci Data.

[14] Hadley D (2017). Precision annotation of digital samples in NCBI’s gene expression omnibus. Sci Data.

[15] McCall MN, Bolstad BM, Irizarry RA (2010). Frozen robust multiarray analysis (fRMA). Biostatistics.

[16] Davis S, Meltzer PS (2007). GEOquery: a bridge between the Gene Expression Omnibus (GEO) and BioConductor. Bioinformatics.

[17] Gautier L, Cope L, Bolstad BM, Irizarry R (2004). A. affy–analysis of Affymetrix GeneChip data at the probe level. Bioinformatics.

[18] Becht E (2019). Dimensionality reduction for visualizing single-cell data using UMAP. Nat Biotechnol.

[19] Leek JT, Storey JD (2007). Capturing heterogeneity in gene expression studies by surrogate variable analysis. PLoS Genet.

[20] Carvalho BS, Irizarry RA (2010). A framework for oligonucleotide microarray preprocessing. Bioinformatics.

[21] Calza S (2007). Filtering genes to improve sensitivity in oligonucleotide microarray data analysis. Nucleic Acids Research.

[22] Colaprico A (2016). TCGAbiolinks: an R/Bioconductor package for integrative analysis of TCGA data. Nucleic Acids Research.

[23] Risso D, Schwartz K, Sherlock G, Dudoit S (2011). GC-content normalization for RNA-Seq data. BMC Bioinformatics.

[24] Wickham, H. *Ggplot2: elegant graphics for data analysis. *R package version 3.3.3. (2016).

[25] M Tsagris & M Papadakis. Forward regression in R: from the extreme slow to the extreme FAST. *J Data Sci* **16**, 771–780 (2018).

[26] Tran PMH (2020). Comparative analysis of transcriptomic profile, histology, and IDH mutation for classification of gliomas. Sci Rep.

[27] Hahsler, M., Piekenbrock, M. & Doran, D. dbscan: Fast density-based clustering with *R*. *J* *Stat Soft* **91**, 1–30 (2019).

[28] Hennig, C. *fpc: Flexible Procedures for Clustering.* R package version 2.2.9. (2020).

[29] Chen H, Boutros PC (2011). VennDiagram: a package for the generation of highly-customizable Venn and Euler diagrams in R. BMC Bioinformatics.

[30] Hänzelmann S, Castelo R, Guinney J (2013). GSVA: gene set variation analysis for microarray and RNA-seq data. BMC Bioinformatics.

[31] Alexa, A., & Rahnenfuhrer, J. *topGO: Enrichment Analysis for Gene Ontology.* R package version 2.42.0. (2020).

[32] Rohr M (2021). Express.

[33] Rohr M (2021). figshare.

[34] Galamb O (2007). Gene Expression Omnibus.

[35] Sabates-Bellver J (2007). Gene Expression Omnibus.

[36] Hong Y, Downey T, Eu WK, Koh PK, Cheah PY (2010). Gene Expression Omnibus.

[37] Galamb J (2010). Gene Expression Omnibus.

[38] Skrzypczak M (2010). Gene Expression Omnibus.

[39] Tsukamoto S (2011). Gene Expression Omnibus.

[40] Okazaki S (2012). Gene Expression Omnibus.

[41] Olivero M (2013). Gene Expression Omnibus.

[42] Uddin S (2011). Gene Expression Omnibus.

[43] Khamas A (2012). GEO.

[44] de Sousa E Melo F (2011). Gene Expression Omnibus.

[45] Galamb O (2012). Gene Expression Omnibus.

[46] Shi X (2015). Gene Expression Omnibus.

[47] Badic B (2020). Gene Expression Omnibus.

[48] Getz G, Gal H, Kela I, Notterman DA, Domany E (2003). Gene Expression Omnibus.

[49] Kalmár A (2019). Gene Expression Omnibus.

[50] Reumers J (2018). Gene Expression Omnibus.

[51] Greytak SR, Engel KB, Bass BP, Moore HM (2015). Accuracy of molecular data generated with FFPE biospecimens: Lessons from the literature. Cancer Res.

[52] American Cancer Society. Colorectal cancer facts & figures 2017–2019. *American Cancer Society* (2017).

[53] Ai, D., Wang, Y., Li, X. & Pan, H. Colorectal cancer prediction based on weighted gene co-expression network analysis and variational auto-encoder. *Biomolecules* **10**, 1207 (2020). 10.3390/biom10091207 PMC7563725 32825264

[54] Shen X (2016). Microarray analysis of differentially-expressed genes and linker genes associated with the molecular mechanism of colorectal cancer. Oncol Lett.

[55] Hauptman N, Glavač D (2017). Colorectal cancer blood-based biomarkers. Gastroenterol Res Pract.

[56] Druliner BR (2018). Molecular characterization of colorectal adenomas with and without malignancy reveals distinguishing genome, transcriptome and methylome alterations. Sci Rep.

[57] Jung B, Staudacher JJ, Beauchamp D (2017). Transforming Growth Factor β superfamily signaling in development of colorectal cancer. Gastroenterology.

[58] Weng W (2016). FOXM1 and FOXQ1 are promising prognostic biomarkers and novel targets of tumor-suppressive miR-342 in human colorectal cancer. Clin Cancer Res.

[59] Rochlitz CF, Herrmann R, de Kant E (1996). Overexpression and amplification of c-*myc* during Progression of Human Colorectal Cancer. Oncology.

[60] Crotti S (2017). Extracellular matrix and colorectal cancer: How surrounding microenvironment affects cancer cell behavior?. J Cell Physiol.

[61] Albasri AM, Elkablawy MA, Ansari IA, Alhujaily AS, Khalil AA (2019). The prognostic significance of p63 cytoplasmic expression in colorectal cancer: An immunohistochemical study. SMJ.

[62] Ohuchi M (2018). Increased EZH2 expression during the adenoma-carcinoma sequence in colorectal cancer. Oncol Lett.

[63] Wang H, Birkenbach M, Hart J (2000). Expression of Jun family members in human colorectal adenocarcinoma. Carcinogenesis.

[64] Evdokimova V, Tognon C, Ng T, Sorensen PHB (2009). Reduced proliferation and enhanced migration: two sides of the same coin? Molecular mechanisms of metastatic progression by YB-1. Cell Cycle.

[65] McLean MH (2011). The inflammatory microenvironment in colorectal neoplasia. PLoS ONE.

